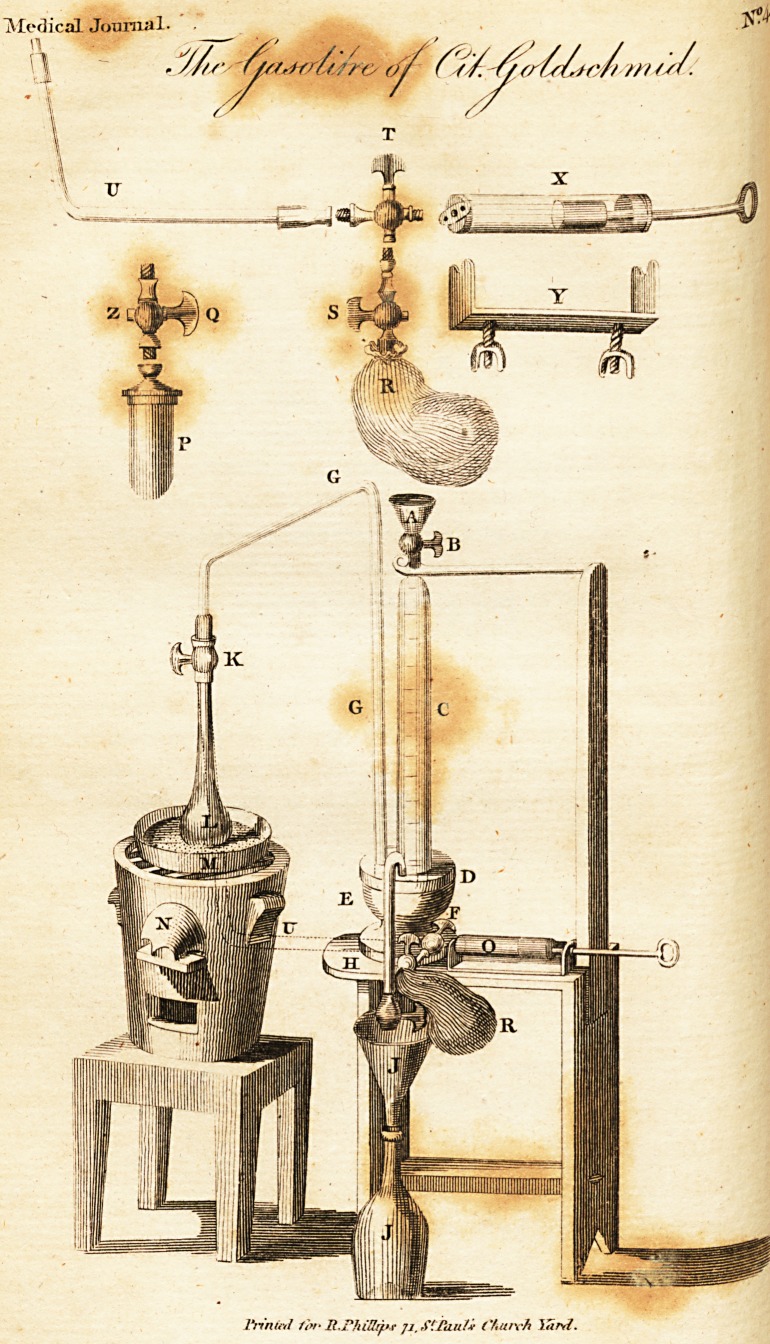# An Account of the Factitious Mineral Waters of Citizen Goldschmid

**Published:** 1799-06

**Authors:** 


### *An Account of the Factitious Mineral Waters of Citizen* GOLDSCHMID.

[*Extracted from the 'Recueil Periodique de la Societé de Médicine de Paris, of Ventôse, An. vii.* (*February*, 1799.)

(Accompanied by an Engraving.)

IN our last Number, p. 296, we announced Citizen GOLDSCHMID's memoir, and the superior virtues which he asserts his artificial Seltzer, Spa, and Sedlitz waters possess over those from the natural springs. We now proceed to lay before our readers, an account of these artificial waters, as contained in a report of Citizens BOUILLON-LA GRANGE, and CHAUSSIER,

CHAUSSIER, read in the Medical Society of Paris, on the 27th Pluviôse (15th February).

By this report it appears, that they examined three distinct species of artificial mineral waters; namely, those of Seltz, Spa, and Sedlitz.

Their first care was to ascertain the physical properties; they found them very clear and transparent, having a strong acid flavour; and that they gave the tincture of turnsol a deep red colour. By putting them in contact with different re-agents, they obtained, first, by caustic alkalis, neutral salts; secondly, by lime water, an abundant carbonate of lime; thirdly, poured on filings of very pure iron, the artificial waters, in a short time, acquired a ferruginous taste, which, after the properties already discovered, confirmed the presence of the carbonic acid and its action on the iron.

They then proceeded to examine the nature of the salts employed, and the quantity of carbonic acid which each bottle might contain. The methods followed to ascertain the composition of these waters, afford no novelty; the saline substances, however, employed, were similar to those described by BERGMANN. Upon the whole it appeared that the author had completely succeeded, and produced an exact imitation of nature.

As to the carbonic acid which these waters possess in solution, experience shews, they have a greater quantity than the natural waters. But in order to ascertain this quantity with the greatest exactness, Citizen Goldschmid has invented a very ingenious apparatus. (See the annexed engraving*) By this apparatus it was discovered, that the waters contained twice and a half their volume of carbonic acid. The author even went so far as to assert, that he could separate three times their volume. But this superabundant quantity easily disengages itself, not only by being agitated, but by an elevation of temperature. Such an excess of acid is therefore useless, especially in medical practice.

The Medical Society also desired the Citizens Bouillon-La Grange, and Chaussier, to examine the difference which existed between the artificial mineral waters of Goldschmid, and those of Citizen GOSSE, of Geneva; but from the impossibility of procuring those of the latter, as recently prepared as those of the former, they were unable to make a fair trial. It is certainly very easy to conceive, that water which has been agitated by travelling,

* This apparatus may serve to obtain any other gas. It possesses the peculiar advantage of preventing the escape of the smallest portion, and, therefore of ascertaining with the most scrupulous exactness the quantity of gas held in a liquid in solution.

velling, or which has been prepared any length of time, whether in a cellar or any other place, will not bear a comparifon with water which has been faturated only a few days. The two commiffioners, therefore, had recourfe to the report made by their colleague, Deyeux, to the School of Medicine, who had carefully examined the artificial water of Citizen Goffe.

"Two pounds" fays that chemift, "of this water, placed under mercury, afforded twice and a half their volume of carbonic acid, the half of which was feparated fpontaneoufly, and the other half by plunging the bottle into hot water."

It is evident from this, that there exifts a great analogy in the refult of the two experiments, fince each of the waters contain twice and a half their volume of carbonic acid; and it is alfo evident, that there is no difference between the mineral waters of Goldfchmid and thofe of Goffe; for, befides the equal quantity of carbonic acid, the chemical examination of the falts in diffolution, afforded the fame refults as thofe mentioned in the report of Citizen Deyeux.

This comparifon muft neverthelefs be of great advantage to thofe who prefcribe or make ufe of thefe artificial mineral waters, as it will enable them to obtain them of a proper degree of acidity; for it is evident, from the experiments of Citizen Goldfchmid, that it is poffible to fuperfaturate the water with the acid.

It appears that thefe artificial waters have been employed with great fuccefs in different maladies by feveral French phyficians; and Bouillon-La Grange, and Chauffier, conclude their report by recommending the eftablifhment of a manufactory for them in Paris, in order to infure their being accurately and faithfully prepared, and alfo by declaring Citizen Goldfchmid deferving both of attention and encouragement.

### *Explanation of the Gafolitre:—By Citizen* Goldschmid.

"THE method hitherto ufed to afcertain the quantity of carbonic acid gas by lime-water, appeared to me very troublefome, not only becaufe it at the fame time precipitates the iron, the magnefia, and other earthy fubftances, which we are afterwards forced to feparate, and becaufe the operation of filtering always occafions a certain wafte, but becaufe the accefs of atmofpheric air contributes, in a great degree, to form carbonate of lime."

"The pneumato-chemical apparatus with mercury, which is at prefent in ufe, has the difadvantage of being always in equilibrium with the preffure of of the atmofpheric air; and is confequently obedient to the laws of the barometer.

"These feveral difficulties urged me to an endeavour at avoiding thofe obftacles, which I have overcome by the invention of the Gafolitre: this apparatus being fully adapted to calculate with accuracy, and in a fhort time, the gafeous parts contained in any body whatever, and to fhew the preffure.

"Fig. P is a glafs meafure containing two centilitres, or two drachms fifty grains: in fitting it with its cock for a double current of air, to the tube G the cylinder C being full of mercury, the cock B fhut, and the apparatus deprived of all poffibility of admitting the atmofpheric air, we fhall find, in opening the cock H that no vacuum is occafioned in the cylinder C befides that which is formed of the bubbles of air between the glafs and the mercury, in filling the cylinder under the preffure of the atmofphere of feventy-five centimetres, or about twenty-eight inches, and that which the difference of the column of mercury in the cylinder C and the volume of air in the meafure P under a preffure of feventy-five centimetres, reduced to zero, can produce.

"We fhall find this ftatement accurate in opening the cock Q to communicate the preffure of the atmofpheric air into the meafure P: in then fhutting it oppofite Z it will not produce a greater vacuum in the cylinder C than the preffure of the volume of air in P has been able to effect.

"The little pump O placed on the ftand F ferves to graduate the cylinder C. We fill the bladder of gas, which we intend to meafure, and open the cock S and T to draw a volume of gas, at all times equal, into the pump; we open the cock T oppofite U fhutting it at the fame time oppofite S and pufh forward the pifton to introduce the gas into the cylinder C filled with mercury, and fhut by the cock B: when the cock H does not fupply more mercury, we continue in this manner to introduce at intervals the fame volume of gas into the cylinder C in order to graduate it.

"We fhall obtain the firft time a greater vacuum on account of the preffure of feventy-five centimetres, where the mercury meets; this preffure ceafes the fecond and following times, when it will be of an equal volume.

"The operation will never be attended with fuccefs, if the external air can penetrate: it is, however, eafy to afcertain this by the cock H which, when the whole is well adjufted and fhut, does not furnifh more mercury after the effort of the preffure before explained.

To

### The Gasolitre of Cit. Goldschmid.

Printed for R. Phillips 71, St. Paul's Church Yard.

To know the quantity of gas which the gaseous waters contain, we thus dispose the apparatus.

"We shut the cock H and fill the cylinder C with mercury; we shut the cocks B and K and open the cock H to ascertain the state of the apparatus; if the apparatus be in proper order, it will not make a vacuum in the cylinder C, farther than the first mark; we fill the matrass up to the neck with the water we intend to analyse, and carefully fit the matrass to the cock K, by a screw provided with a little shield of leather: the whole being well secured, we open the cocks K and H and heat the water to the boiling point."

EXPLANATION OF THE PLATE.

A. Glass funnel to pour in the mercury.

B. Iron cock.

C. Glass cylinder, graduated, shut by the cock, and cemented on the plate D.

D. Delft plate.

E. Wooden stand.

F. Stand to support the apparatus.

G. Glass tube, the cavity of which is of the diameter of one millimetre, about half a line, and which communicates with the cylinder C.

H. Tube of the same diameter, communicating with the cylinder C, and returning into the bottle J by an iron cock.

J. Bottle and funnel to receive the mercury.

K. Cock for a double current of air.

L. Matrass containing about twelve centilitres of water, (four ounces) secured to the cock by a screw.

M. Sand bath.

N. Furnace.

O. Pump, the cylinder of which is glass, secured by two screws to the stand F.

P. Glass measure of two centilitres, or 6 drachms twenty-eight grains.

Q. Cock for a double current of air.

R. Bladder to contain the gas.

S. Cock.

T. Cock for a double current of air.

U. Glass pipe coming from the cock T to K.

X. Pump.

Y. Stand on which to fix the pump.

Z. Index of the second current of air.

*A Comparative*

## Figures and Tables

**Figure f1:**